# Report of Diseases, from the 10th of Semptember to the 10th of December, 1822; Being a List of the Complaints Affecting Children, Treated at the Royal Infirmary for Sick Children

**Published:** 1823-01

**Authors:** John Webster

**Affiliations:** one of the Physicians to that Institution.


### STATISTICAL MEDICINE.

ART. I.—*Report of Diseases, from the* 10*th of Semptember to the* 10*th of December,* 1822; *being a List of the Complaints affecting Children, treated at the Royal Infirmary for Sick Children.* By JOHN WEBSTER, M.D. one of the Physicians to that Institution.

***Diseases affecting particular Organs.***

| | | | | |
|---|---|---|---|---|
| Head and Nervous System | | Hydrocephalus Acutus | 18 | |
| | | Epilepsia | 3 | |
| | | Concussio Cerebri | 1 | = 22 |
| Organs of Respiration | | Hæmoptisis | 1 | |
| | | Tracheitis | 3 | |
| | | Bronchitis | 86 | |
| | | Pneumonia | 5 | |
| | | Tussis | 14 | |
| | | Pertussis* | 81 | =190 |
| Organs of Digestion | Mouth | Aptha | 9 | |
| | Stomach | Dyspepsia | 26 | |
| | Bowels | Constipatio | 3 | |
| | | Diarrhœa | 19 | |
| | | Dysenteria | 10 | |
| | Liver | Hepatitis Chronica | 1 | |
| | | Icterus | 2 | |
| | All three | Cholera | 16 | |
| | General | Marasmus | 3 | |
| | Worms | Lumbricus | 2 | |
| | | Tænia | 1 | |
| | | Ascarides | 12 | =104 |
| Skin Eruptions | Acute | Scarlatina | 24 | |
| | | Variola — Confluens | 1 | |
| | | Variola — Discreto | 3 | |
| | | Varicella | 5 | |
| | | Morbilli | 6 | |
| | | Rubeola | 3 | |
| | | Ecthema Cachecticum | 2 | |
| | | Purpura Hæmorrhagica | 1 | |
| | Chronic | Lichen | 1 | |
| | | Porrigo | 2 | |
| | | Papulæ | 1 | = 49 |

***Diseases affecting various Parts.***

| | | | |
|---|---|---|---|
| Muscles, Tendons, Joints, &c. | Rheumatismus Acutus | | 1 |
| Fevers | Continua Simplex | 121 | |
| | ——— Cephalica | 12 | |
| | ——— Pleuritica | 24 | |
| | ——— Gastro-enteritica | 30 | |
| | Remittens | 43 | =230 |
| Dropsies | Ascites | | 2 |
| Cachexia | | | 6 |
| Vaccinated | | | 9 |
| Names of diseases not mentioned | | | 10 |
| | Total number of patients | | 623 |

* Although Pertussis be placed amongst the diseases affecting the organs of respiration, it is out of deference to the old and generally received opinion, not from a thorough conviction on the part of the author; for on this point he has great doubts, as may be seen by referring to a paper on this disease, in the last Number (for December) of this Journal.

*Fatal Cases reported at the Infirmary.* Diseases:—Bronchitis, 3; Tracheitis, 1; Pertussis, 3; Febris simplex, 1; Feb. Pleuritica, 1; Rubeola, 1; Diarrhœa, 1; Ecthema Cachecticum, 1.—Total, 12.

Although the diseases of children have been both numerous and well marked during the last three months, still they do not comprehend an extensive class of complaints. This may easily be explained, when it is considered that children have generally good constitutions, which, unlike those of adults, have not been injured by intemperance, mental affections, or, in most cases, by previous illness; but are healthy subjects exposed to, or influenced by, comparatively fewer collateral causes of disease: hence their complaints are, in general, distinct, easily understood, and can, for the most part, be successfully treated.

In looking over the above table, any one may naturally be struck at observing so few deaths reported in such a formidable list of diseases; and it will at once be concluded that they cannot by any means be considered as indicating the correct rate of mortality. To explain this circumstance, two things, alike applicable to all medical charities, but peculiarly so to the present one, must not be lost sight of:—namely, that the parents sometimes neglect to inform the medical officers of the death of a patient, and sometimes they conceal it, in the fear lest the body should be opened; a feeling that still operates, particularly amongst the poor, though now not so much as it formerly did. But the chief reason why so few deaths appear to have taken place, is owing to the facility with which relief can be obtained by a simple application at the Infirmary: disease being thus taken in the commencement, little difficulty is met with in their treatment. In acute diseases, this remark is peculiarly applicable; and, for the most part, those which have come under observation have been seen a day or two after the disease has begun: this may account for the few fatal cases reported.

The disease of most frequent occurrence during this trimestre is fever, of which not fewer than 230 cases have been treated. It was generally of a mild type, and yielded usually to purgatives, saline medicines, and diluents. Local bleeding by leeches was also employed, where any particular organic affection existed; but general bleeding was only had recourse to in one instance, and that proved a very tedious one. In the treatment of children, particularly those of the poorer classes, one of the great objects to be kept constantly in view is, not only to do what may be useful, but to prevent the friends of the patient from doing that which is hurtful; for they have an idea that the strength of the patient must be supported against the weakening effects of fever: on this account, wine, spirits, porter, and nourishing food, are given. Many of the worst cases that occurred were chiefly owing to a non-observance of the diet and regimen laid down. Even during convalescence from fever, nothing ought to be more carefully guarded against than the giving of improper food; and the slow recoveries often met with, very much depend on inattention to this rule. In the above table, continued fever has been classed under four separate heads. This is an arbitrary division, but its object is to show what were the most prominent symptoms of the disease. In the Febris continua simplex, there existed no particular apparent organic affection; the Cephalica shows that the head was principally affected; the Pleuritica, the chest; and the Gastro-enteritica, the stomach and bowels. In the early part of the period above alluded to, the common continued fever almost entirely prevailed; the pleuritic and cephalic varieties in October and the early part of November; and lately the gastro-enteritic was almost the only kind that occurred. The average of remittent fever has nearly been the same during the whole of this period.

Bronchitis has been a very common disease, especially in the month of November and the early part of December: it was in general of a mild kind, and, when severe, principally in very fat children. The three fatal cases reported were all in very corpulent subjects. This leads to the remark, verified by repeated observation, that, the fatter the child affected with bronchitis is, the more unfavourable ought the prognosis to be: the more so, as they do not bear bleeding and depletion so well as those children do who are of a more spare habit of body. Leeches, frequent and small doses of calomel and ipecacuanha or rhubarb,—tartarized antimony, given in solution, so as to keep up a constant nausea,—blisters, and the warm bath, were the common modes of treatment; and were found very beneficial, nothing appearing to give more relief, for the time at least, than the bath,—the chest and neck being always immersed in the warm water.

A great number of cases of Hooping-cough have been met with during the above period, and many of them were of long standing. The treatment recommended in the last Number of this Journal has been put in practice very extensively, and with the most decided benefit. One case may be mentioned, in which the method therein spoken of was tried. The child was two years and three months old, and had been ill of hooping-cough for upwards of four weeks. When first seen, the mother said it had from three to four severe fits in an hour; it was then feverish, and much exhausted by the disease. Three leeches were applied to the forehead, and a dose of calomel, rhubarb, and nitre, was ordered to be given every night at bed-time. On the third day after, the cough was better, and hooping much less severe. On the 7th, had only hooped five times the preceding twenty-four hours, and was otherwise much better. Another leech was applied to the forehead, and a purging mixture, with syrup of squills, prescribed at the last visit, was continued. On the fourteenth day after admission, the report was, "Does not hoop at all; bowels open; and only a little feverish at night." —R. Pulv. Cort. Cinchonæ, Sodæ Carbonatis, āā gr. iij. ter die, inter alia.—A week afterwards, the child, being quite well, was discharged.

Many other corroborating examples might be adduced, if necessary, from the eighty-one cases which occurred, showing the utility of this treatment; but it will suffice to remark, that leeches to the forehead was generally the chief, often the only, remedy depended upon, especially in the early stages of the disease.

Hydrocephalus, being generally seen in the commencement of the disease, was not often very dangerous, though several very acute cases were met with. Leeches, calomel, purgatives, blisters to the nape of the neck, cold applications to the head, and diuretic medicines, were the remedies usually employed. Of these, the most benefit appeared to follow the use of diuretics, especially after bleeding and purging had been actively carried into effect. At least, it was certainly found that, the more freely the kidneys secreted, the more benefit seemed to be obtained. The medicines which were commonly prescribed with this view were squills, nitrous ether, and digitalis.

Of the twelve cases which proved fatal, seven were examined. The most interesting was that of Tracheitis: in it the trachea was found nearly filled with the tubular membrane which occurs in that disease. It was not equally firm in all its parts, but it formed a very beautiful specimen of the termination of this complaint. The subject was a girl, of about three years of age, and was somewhat corpulent. The disease, it was said, had existed about three weeks when brought to the Infirmary; but little could then be done, as the disease appeared near its termination, and the patient died next day. The rattling noise in the throat, peculiar to this disease, was very distinctly marked, and the child was suffocated by the membrane now spoken of.

Three cases of Bronchitis were examined: in all, there was considerable redness on the internal surface of the trachea and bronchiæ; in one, the inflammation had extended to the pleura; and, in all, death appeared to be produced by the accumulation of a mucous matter in the bronchia, especially at the bifurcation of the trachea, thus producing suffocation.

A case of Hooping-cough showed no evident marks of disease in the organs of respiration, but considerable congestion in the head, and a slight effusion of lymph-like matter on the pia mater, with a small portion of water in the ventricles.

The fatal case of Fever was examined: in it, two or three ulcerated spots on the mucous membrane of the small intestines were the only morbid appearances met with.

The remaining case which was examined was that of the Ecthema Cachecticum: there the lungs were very much diseased, being quite filled with tubercles, some of which had already gone on to ulceration. The whole surface of the spleen was covered with small specks of a yellowish colour; a few also appeared to be on the surface of the small intestines, but whether of the same nature as those on the skin cannot be asserted, as the same appearances on the spleen have before been met with in a child, a patient at the Infirmary, who died of quite another disease, in the early part of last summer.

N. B.—The number of patients given in the foregoing table includes only those treated by Dr. Webster, exclusive of the other medical officers; the total number of children admitted on the books of the Institution during the three months amounting to 1,830.

